# Ablation index-guided cavotricuspid isthmus ablation with contiguous lesions using fluoroscopy integrated 3D mapping in atrial flutter

**DOI:** 10.1007/s10840-022-01182-4

**Published:** 2022-03-16

**Authors:** Susumu Sakama, Atsuhiko Yagishita, Tetsuri Sakai, Masahiro Morise, Kengo Ayabe, Mari Amino, Yuji Ikari, Koichiro Yoshioka

**Affiliations:** Department of Cardiology, Tokai University, Shimokasuya 143, Isehara, Kanagawa Japan

**Keywords:** Cardiac arrhythmia, Atrial flutter, Cavotricuspid isthmus ablation, Ablation index, CARTO UNIVU

## Abstract

**Purpose:**

The feasibility and safety of cavotricuspid isthmus (CTI) ablation with contiguous lesions using ablation index (AI) under the guidance of fluoroscopy integrated 3D mapping (CARTO UNIVU/CU) in typical atrial flutter (AFL) remains uncertain. This study aimed to determine the efficacy of AI-guided CTI ablation with contiguous lesions in patients with AFL.

**Methods:**

In this single-center, prospective, non-randomized, single-arm, observational study, procedural outcomes were determined in 151 patients undergoing AI-guided CTI ablation (AI group) with a target AI value of 450 and an interlesion distance of ≤ 4 mm under CU guidance. These outcomes were compared with those of 30 patients undergoing non-AI-guided ablation (non-AI group).

**Results:**

Among 151 patients, first-pass conduction block was achieved in 120 (80%) patients in the AI group (67% in the non-AI group, *P* = 0.152) with a shorter fluoroscopy time of 0.2 ± 0.4 min (1.7 ± 2.0 min in the non-AI group, *P* < 0.001). Conduction gaps were located at the atrial aspects near the inferior vena cava in 24 of 31 (78%) patients without first-pass conduction block. The AI group received 11 ± 5 (12 ± 4 in the non-AI group, *P* = 0.098) radiofrequency (RF) applications, and the RF time was 4.2 ± 2.4 (5.1 ± 2.5 min in the non-AI group, *P* = 0.011). Despite the occurrence of steam pop in 3 (2%) patients, none of them developed cardiac tamponade. No patients had recurrence within 6 months of follow-up.

**Conclusions:**

AI-guided CTI ablation in combination with CU was feasible and effective in reducing radiation exposure in patients with AFL.

## Introduction

Cavotricuspid isthmus (CTI)-dependent atrial flutter (AFL) is a common arrhythmia that causes heart failure and thromboembolic events. Slow conduction of an atrial tissue between the tricuspid annulus and the inferior vena cava with conduction block by anatomical boundaries of the crista terminalis and Eustachian ridge is an electrophysiological substrate of the AFL. Radiofrequency (RF) catheter ablation of the CTI is an established therapy for AFL [1].

Ablation index (AI, CARTO 3, Biosense Webster, Inc., Diamond Bar, CA) is a method that incorporates contact force, time, and power in a nonlinear formula in real time [2, 3]. In patients with atrial fibrillation (AF), AI-guided pulmonary vein isolation (PVI) aiming to enclose the veins achieves a higher rate of freedom from recurrence [4, 5]. Furthermore, a recent study demonstrated that contiguous lesions with a shorter interlesion distance (3.0–4.0 mm) were associated with a higher first-pass PVI than those with an interlesion distance of 5.0–6.0 mm [6]. Several studies have reported the utility of AI for CTI ablation [7, 8]; however, an optimal AI target for effective CTI ablation remains uncertain, particularly in cases with a shorter interlesion distance (3.0–4.0 mm). CARTOUNIVU (CU, CARTO 3, Biosense Webster, Inc.) integrates fluoroscopic images into a three-dimensional (3D) electroanatomical map and allows accurate catheter manipulation without using fluoroscopy, resulting in the reduction of fluoroscopic use [9]. To date, no studies have examined the efficacy of the combined use of AI and CU in CTI ablation for AFL. In this study, we aimed to determine the efficacy of AI-guided CTI ablation with contiguous lesions in patients with AFL.

## Methods

### Ethical information

This study complied with the Declaration of Helsinki regarding investigation in humans and was approved by the Institutional Ethics Committees at Tokai University Hospital (20R-392, April 26, 2021).

### Study population

This single-center, prospective, non-randomized study included 151 consecutive patients who underwent AI-guided CTI ablation under the guidance of CU for AFL. Patients with any condition contraindicating chronic anticoagulation, active systemic infection, pregnant or breastfeeding women, or women of childbearing potential not on adequate birth control were excluded. Baseline clinical parameters, including CHA2DS2-VASc [congestive heart failure, hypertension, age ≥ 75 (doubled), diabetes mellitus, prior stroke or transient ischemic attack (doubled), vascular disease, age 65–74, female] score, and procedural outcomes, including the rate of first-pass conduction block across the CTI, duration of fluoroscopic use from the start of CTI ablation until the completion of conduction block, number of RF applications, delivered energy, and complications were determined and compared with those of patients who underwent CTI ablation without using AI and CU (*n* = 30, non-AI group) during the same period of enrollment of those with AI-guided CTI ablation.

### Ablation procedure and follow-up

**Fig. 1:**
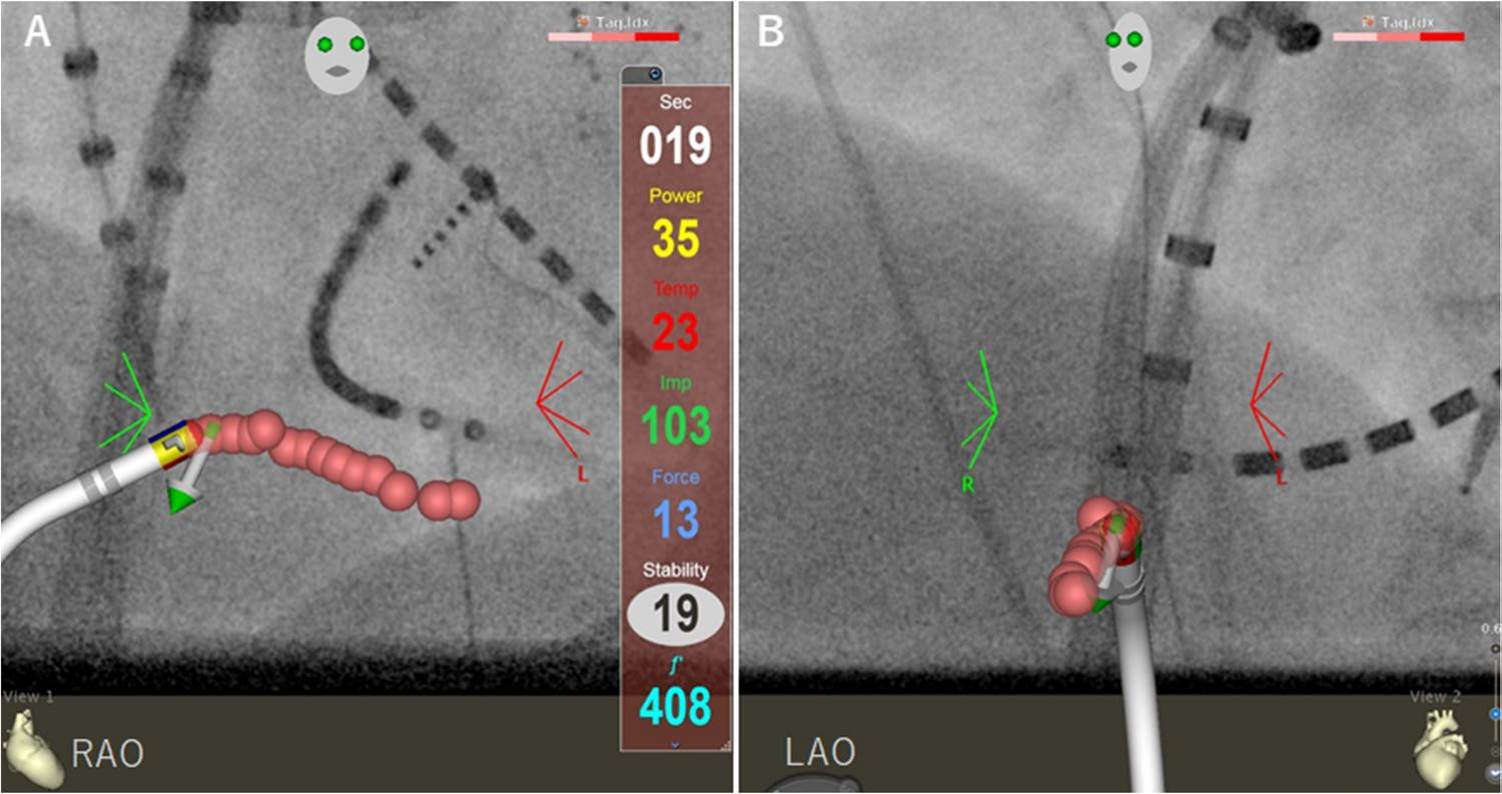
Ablation index-guided cavotricuspid isthmus ablation in fluoroscopy integrated 3D mapping. Cavotricuspid isthmus ablation with contiguous lesions using ablation index (AI) under the guidance of CARTO UNIVU (**A**, right anterior oblique view; **B**, left anterior oblique view) in a patient with typical atrial flutter. The pink dots represent ablation lesions with a diameter of 4 mm and an interlesion distance of ≤ 4 mm, and the contiguous lesions using AI are shown in a light blue color on the screen

The absence of atrial thrombus was confirmed by transesophageal echocardiography or computed tomography [10]. The procedures were performed under conscious sedation and uninterrupted oral anticoagulation. Right femoral venous access was obtained, and a steerable duo-decapolar electrode catheter (6F BeeAT, Japan Lifeline Co. Ltd. Tokyo, Japan) was introduced into the coronary sinus (CS), thereafter allowing simultaneous recording of the right atrium (RA). A contact-force (CF)-sensing ablation catheter (Thermocool SmartTouch STSF, Biosense Webster, Inc.) was introduced via the right femoral vein through a steerable Agilis NxT (Abbott Laboratories, Abbott Park, IL) or VIZIGO (Biosense Webster, Inc.) sheath into the RA, and a 3D electroanatomical mapping system (CARTO, Biosense Webster, Inc.) was used for ablation (Fig. 1). In patients with sinus rhythm, catheter ablation was performed during pacing from the proximal CS at 600–800 ms. In patients with AFL, catheter ablation was completed to achieve bidirectional conduction block during pacing from the CS following termination of AFL. Point-by-point ablation was performed from the tricuspid valve annulus to the inferior cava veins. The VISITAG module was used to mark the location of each lesion during RF applications, and the settings were as follows: stability range of 2–3 mm, stability time of 3–5 s, force over time of 25%, 3-g force, and a tag size of 4 mm (diameter) [6]. The power setting was 35 W with a CF of 5–30 g, AI target value of 450 [7], and an interlesion distance of < 4 mm (Fig. 1). Saline irrigation and temperature limit were set at 30 mL/min and 43 °C, respectively. RF was applied at the same spot where the catheter instability did not reach the target value. First-pass conduction block was defined as a bidirectional conduction block through the CTI either before or after the linear ablation without additional RF applications, which was confirmed by a separate double potential at the distal bipoles of an ablation catheter during the CS pacing and differential pacing maneuvers [11]. In cases with no first-pass conduction block, the location of the conduction gaps was identified to achieve a bidirectional conduction block with additional RF applications, and intracardiac echocardiography (ICE) was performed to assess the presence of the sub-Eustachian pouch at the location of the conduction gaps [12]. Isoproterenol (20–30 μg/min) was administered intravenously during a waiting time of 15 min [13]. In cases with reconnection, the location of the conduction gaps, where additional RF applications achieved bidirectional conduction block, was identified.

In the non-AI group, conventional CTI ablation was performed using a CF-sensing irrigated catheter with a power setting of 35 W and 30 s at each point with a CF of 5–30 g until bidirectional conduction block was achieved; the Ensite system (Precision, Abbott, Abbott Park, IL) was used in 24 patients, and the Rhythmia™ system (Boston Scientific, Marlborough, MA) was used in six patients. After the procedure, continuous electrocardiogram (ECG) monitoring was maintained for 24 h, and 12-lead ECG was recorded during hospitalization. Follow-up visits were scheduled at 1, 3, and 6 months. Clinical evaluation, 12-lead ECG, and 24-h ambulatory monitoring were performed at 3 and 6 months after the procedure.

### Statistical analysis

Categorical variables were analyzed using the chi-square statistics and expressed as percentages. Quantitative variables were presented as means with standard deviation or as median with interquartile range (IQR), as appropriate for their distribution. The Mann–Whitney U test was used to compare quantitative variables. A two-tailed *P* value of < 0.05 was considered significant. All statistical analyses were performed using the SPSS Statistics (version 20.0; IBM Corp., Armonk, NY, USA).

## Results

### Patient characteristics

The baseline characteristics of the patients are summarized in Table 1. The median age was 69 (IQR 59–74) years, and the CHA2DS2-VASc score was 2 (1–3). There were no differences in the baseline characteristics between the two groups.

**Table 1:** Characteristics of the patients at baseline

	All (*n* = 181)	AI group (*n* = 151)	Non-AI group (*n* = 30)	*P* value
Median age (IQR) years	70 (61–74)	69 (59–74)	72 (66–78)	0.085
Female sex [*n*(%)]	48 (27)	40 (27)	8 (27)	1.000
Median body mass index (IQR) kg/m^2^	23.7 (21.3–26.0)	23.8 (21.3–25.9)	23.3 (21.1–27.5)	0.829
Hypertension [*n*(%)]	88 (49)	74 (47)	14 (47)	0.844
Diabetes [*n*(%)]	34 (19)	28 (19)	6 (20)	0.803
Heart failure [*n*(%)]	26 (14)	22 (15)	4 (13)	1.000
Prior thromboembolic events [*n*(%)]	11 (6)	10 (7)	1 (3)	0.694
CHADS2 (IQR)	1(0–2)	1(0–2)	1(0–2)	0.903
CHA2DS2-VASc (IQR)	2(1–3)	2(1–3)	2(1–3)	0.240
Median estimated GFR(IQR) mL.min^−1^.1.73 m^−2^	56.0 (48.0–67.0)	56.0 (49.0–66.0)	54.5 (42.5–69.3)	0.521
Median BNP level (IQR) pg/mL	89.4 (41.7–178.4)	89.4 (39.6–178.3)	86.0 (45.1–203.3)	0.514
Median left atrial diameter (IQR) mm	40 (37–44)	40 (37–44)	41 (34–45)	0.977
Median left ventricular ejection fraction (IQR) %	65 (59–72)	65 (58–71)	65 (61–73)	0.870

*BNP*, brain natriuretic peptide; *CHADS2*, [congestive heart failure, hypertension, age > 75 years, diabetes (all 1 point each), previous stroke (2 points)]; *CHA2DS2-VASc*, [congestive heart failure, hypertension, age (> 65 = 1 point, > 75 = 2 points), diabetes, previous stroke/transient ischemic attack (2 points)]; *GFR*, glomerular filtration rate; *IQR*, interquartile range; *AI*, ablation index

### Procedural outcomes

**Fig. 2:**
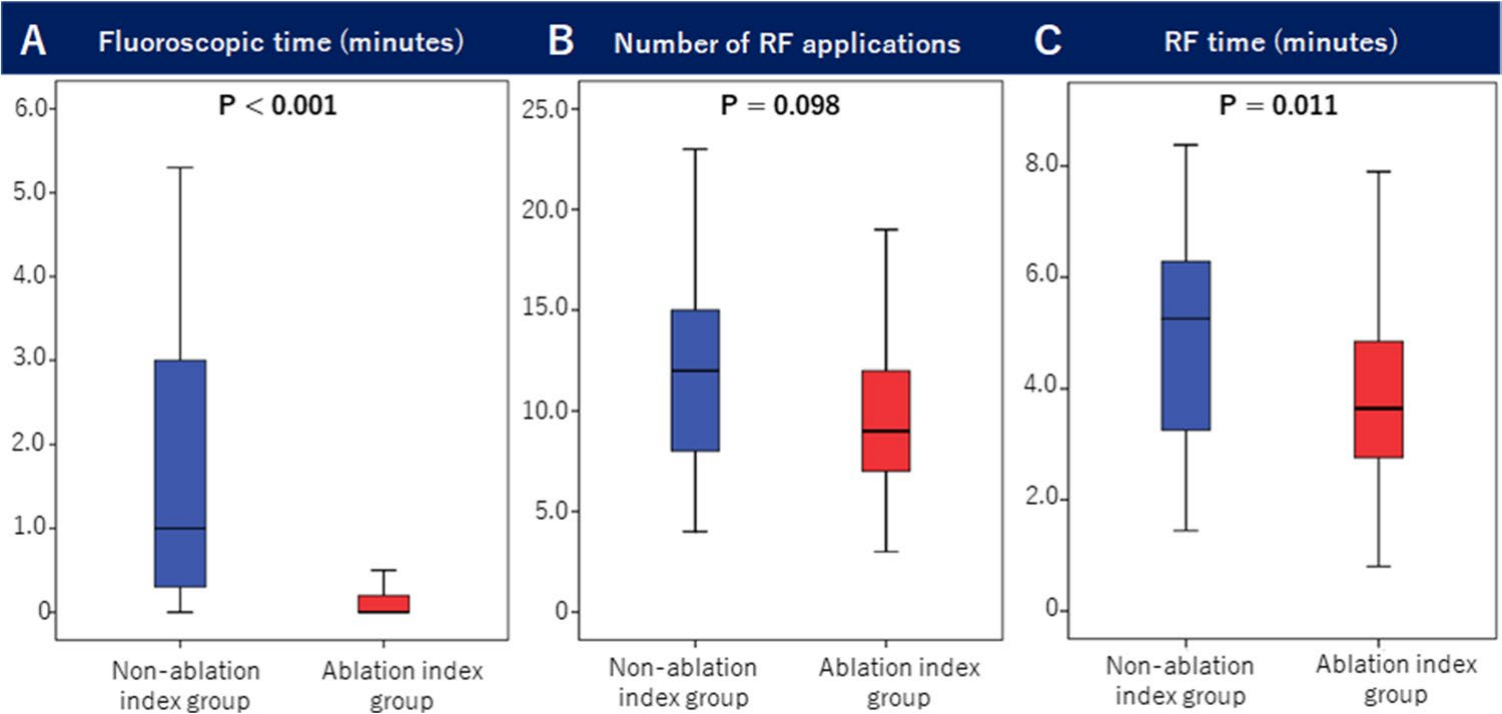
Ablation parameters in patients who underwent ablation index-guided cavotricuspid isthmus ablation. **A** The fluoroscopy time was significantly shorter in patients who underwent ablation-index guided ablation (0.2 ± 0.4 vs. 1.7 ± 2.0 min, *P* < 0.001). **B** The number of radiofrequency (RF) applications was similar between the two groups (11 ± 5 vs. 12 ± 4, *P* = 0.098). **C** The RF time was shorter in patients who underwent ablation index-guided ablation (4.2 ± 2.4 vs. 5.1 ± 2.5, *P* = 0.011)

**Fig. 3:**
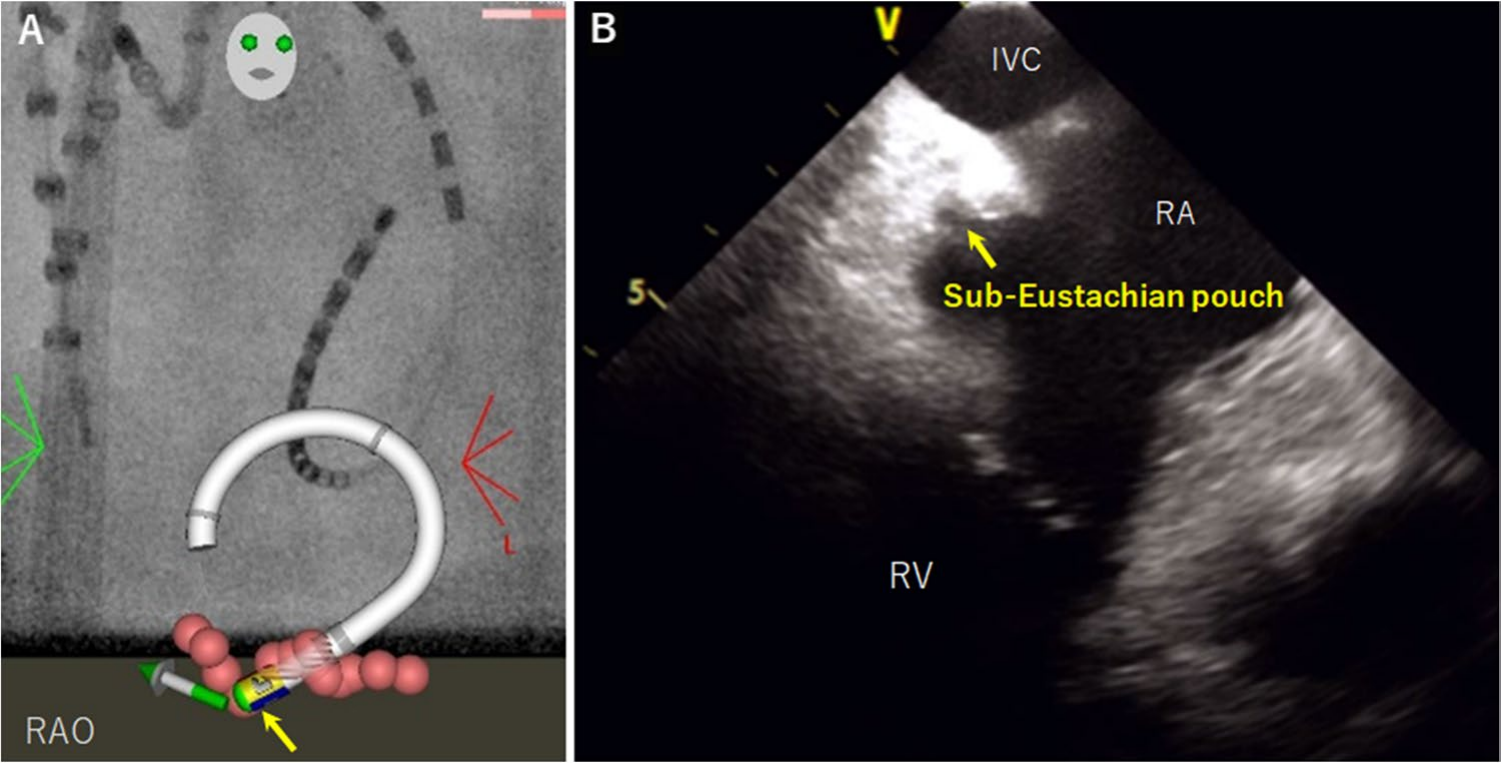
Representative case requiring additional ablation because of a sub-Eustachian pouch. **A** Right anterior oblique (RAO) projection of CARTO UNIVU in a patient where the first-pass conduction block was not achieved. Additional radiofrequency application with an ablation catheter arched over the Eustachian ridge achieved a bidirectional conduction block. **B** Intracardiac echocardiography revealed a sub-Eustachian pouch where additional radiofrequency was applied (yellow arrow). IVC, inferior vena cava; RA, right atrium; RV, right ventricle

The first-pass conduction block was achieved in 120 of 151 (80%) patients, which was 67% in the non-AI group (*P* = 0.152, Fig. 2). The fluoroscopy time was 0.2 ± 0.4 min in the AI group, which was shorter than that in the non-AI group (1.7 ± 2.0 min, *P* < 0.001). In 31 of the 151 patients without the first-pass conduction block, conduction gaps were located at the atrial aspects near the inferior vena cava in 24 (78%) patients. ICE revealed a sub-Eustachian pouch in 8 of the 24 (33%) patients (Fig. 3). The AI group received 11 ± 5 RF applications (12 ± 4 in the non-AI group, *P* = 0.098), and the total RF time was 4.2 ± 2.4 min (5.1 ± 2.5 min in the non-AI group, *P* = 0.011). Reconnection during the waiting time occurred in 16 of 151 (11%) patients, and among these, first-pass conduction block was achieved in 8 (50%) patients. Reconnected gaps were located at the atrial aspect in 11 of 16 (69%) patients. Although steam pop occurred in 3 (2%) patients, none of these developed cardiac tamponades. None of the patients had AFL recurrence after 6 months of follow-up in both groups.

## Discussion

Our major findings were as follows: (1) AI-guided CTI ablation with contiguous lesions under CU guidance achieved first-pass conduction block with shorter fluoroscopy and RF time; (2) the majority of the conduction gaps were located at the atrial aspects of the CTI in patients without a first-pass conduction block, and ICE demonstrated a sub-Eustachian pouch in one-third of them; (3) steam pop occurred in 2% of the patients, but none of them had cardiac tamponade; and (4) none of the patients had AFL recurrence within 6 months of follow-up after the AI-guided CTI ablation.

AFL is a common arrhythmia that causes heart failure and thromboembolic events [14, 15]. Catheter ablation at the CTI between the tricuspid annulus and the inferior vena cava is safe and effective [1]. A previous prospective randomized study compared pharmacotherapy with catheter ablation in patients with AFL and demonstrated that catheter ablation was associated with a higher rate of restoration of sinus rhythm, lower readmission rate, lower incidence of AF, and improvement in the quality of life [16].

AI-guided PVI with contiguous lesions enclosing the veins has been associated with a higher rate of first-pass isolation and freedom from recurrence of AF [4, 5]. The optimal AI for safe and effective CTI ablation is not well established [7, 8]. Zhang et al. demonstrated the feasibility of AI-guided ablation of the CTI line for AFL, where the AI target was set at 500 for the anterior 2/3 segments and 400 for the posterior 1/3 segments of CTI with an interlesion distance of ≤ 6 mm [7]. Compared with the conventional CF-guided catheter ablation, the AI-guided CTI ablation resulted in a higher rate of first-pass conduction block; however, the optimal anatomical boundary between the anterior and posterior CTI was unclear, and the fluoroscopy time was > 3 min in their study. A recent study reported that contiguous lesions with a shorter interlesion distance of 3.0–4.0 mm achieved higher first-pass isolation during PVI than those with an interlesion distance of 5.0–6.0 mm, despite the lower target AI in the closer interlesion distance protocol [6]. In our study, the lower AI target of 450 at the ventricular segments of the CTI (interlesion distance of ≤ 4 mm) achieved a high rate of first-pass conduction block without increasing the incidence of a conduction gap, suggesting that a closer interlesion distance with a lower target AI may allow for less extensive ablation at the ventricular segments of the CTI. Furthermore, we demonstrated the efficacy of the combined used of AI and CU modules for a significant reduction in the use of fluoroscopy in CTI ablation for AFL.

ICE is an important tool for catheter ablation to determine the orientation and tissue contact of an ablation catheter, which ensures proper lesion formation while minimizing the potential for inducing steam pops. In this study, ICE visualized the sub-Eustachian pouch in one-third of the patients without a first-pass conduction block. In a previous study by Regoli et al., a deep right atrial pouch, detected by transesophageal 3D echocardiography, was associated with a significantly prolonged ablation time to achieve a bidirectional block of the CTI [17]. Routine pre-procedural ICE imaging to characterize CTI anatomy may be helpful for further improvement of AI-guided CTI ablation. Moreover, the detection of intramural microbubbles on the ICE during RF applications is an early indicator of steam pop [18]. In this study, steam pop occurred in a minority of patients undergoing AI-guided CTI ablation; however, none of the patients had pericardial effusion or tamponade. Considering that the AI value is not a reliable predictor of steam pop, real-time visualization of intramural microbubbles during CTI ablation may decrease the incidence of steam pop [19].

## Limitations

This study has some limitations. First, it was a small, single-center, observational cohort study. Furthermore, the difference in the number of patients between two groups may have been associated with no statistical difference in the number of RF applications. Second, it is likely that some of the patients we considered free from AFL recurrences might have undetected AFL in the absence of continuous monitoring devices. Third, although ICE imaging was useful to characterize CTI anatomy, cost effectiveness of the use of ICE in CTI ablation has not been validated in this study. Fourth, efficacy of the AI-guided CTI ablation has not been compared with other strategies including maximum voltage-guided CTI ablation [20, 21]. Finally, the lack of systematic invasive procedures to assess reconnections of the CTI and the relatively short follow-up duration of 6 months might have overestimated the efficacy of AI-guided CTI ablation. Therefore, a prospective multicenter study comparing the AI-guided strategy with other ablation strategies with larger sample size and long-term follow-up using continuous monitoring devices such as an implantable loop recorder is required for a detailed analysis.

## Conclusions

AI-guided CTI ablation with contiguous lesions had higher efficacy, with a significant reduction in radiation exposure under the guidance of CU in patients with AFL.
